# 

**Published:** 1857-04

**Authors:** 


### SANITARY REVIEW,

AND

### Journal of Public Health.

EDITED BY

BENJAMIN W. RICHARDSON, M.D.,
LICENTIATE OF THE ROYAL COLLEGE OF PHYSICIANS,
PHYSICIAN TO THE ROYAL INFIRMARY FOR DISEASES OF THE CHEST, AND LECTURER
ON PUBLIC HYGIENE AT THE GROSVENOR PLACE MEDICAL SCHOOL.

NATIONAL HEALTH

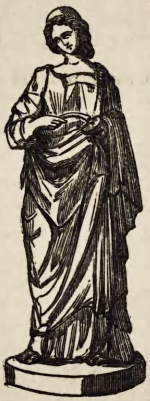

IS NATIONAL WEALTH.

ὙΓΙΕΙΑ

*πρεσβίστη μακάρων.*

VOL. III.

PRINTED AND PUBLISHED FOR THE PROPRIETOR BY
THOMAS RICHARDS, 37, GREAT QUEEN STREET.
1857.

WI
SA672P

T. RICHARDS, PRINTER, GREAT QUEEN STREET.

0951

### INDEX.

Animal food, diseased, 104, 125
Animal physics, 137
British Juggernaut in India, 217
Cardiff, sanitary state of, 392
Caries of the teeth, 13
Chemicals, sanitary improvements in manufacture of, 82
Cholera in lower animals, 263; excessive atmospheric pressure the cause of, 264
Climate and rheumatism, 140
Colliers, Mr. W. I. Cox on diseases of, 48
Copper-smoke and its effects, 14
Crime, legalised causes of, 139
Crimean Sanitary Commissioners' Report, 315
Disinfection and deodorisation, report on, 214
Drainage of London, Dr. Hawksley on, 28; Mr. Nicholas on, 65
Drowning, apparent, treatment of, 192
Epidemic and endemic diseases, local reports of, 84, 192, 297, 398
Ergotised rye, detection of, 83
Fæcal refuse of London, Dr. Ayres on disposal of, 17
Female Sanitary Society, proposal for, 296
Fever, treatment of by fresh air, 262
Food-grains of India, 395
Gas nuisances and their removal, 191
Graveyard statistics of Warwick and Leamington, Dr. J. Webster on, 63
Health of London during 1856, Dr. J. Webster on, 277
Huddersfield, sanitary condition of, 73, 178
Hydrophobia, Dr. Pickells' notes on, 371
Insanity statistics, 139
Life assurance, 263
Measle of the pig, 15
Medical scientific reform, 1
Medical geography and endemic diseases, 249
Medical Pilgrim's progress, Dr. B. W. Richardson on, 353
Meteorological report, 14
Meteorology, Mr. Glaisher's treatise on, 15
Meteorology and epidemics of the seventeenth century, Dr. Winn on, 55
Metropolitan hygiene in the past, 335
Mourning, summer, for females, Dr. F. J. Brown on, 286
Netley Hospital, 316
Nursery government in its sanitary aspects, Dr. H. Barker on, 164
Oesterlen's Handbook of Hygiene, 260
Pauperism in England and Wales, 83
Plague at Dunster in 1645, 10
Post Office, Dr. Lewis's report on health of officers of, 37
Questions of the day, 261
Registration of disease in England, 317
Reports on cities, towns, and districts, 73, 178, 288, 392
Russian army, medical organisation of, 104

42913

Sanitary legislation a first duty of a Government, Mr. Daniel on, 157; notes of, 104, 213, 315, 416; and social science, 73, 178, 288, 392
Scarlet fever, treatment of, 13
Seaweeds, edible, 79
Sewerage and agriculture, 133; metropolitan and town, 136
Sick room, management of, 138
Social epidemic, the, 327
Sweating sickness in England, 105
Teignmouth, sanitary state of, 288
Torquay a resort for consumptives, 137
Turkish army, Mr. J. N. Radcliffe on hygiene of, 141, 265
Vaccination, 239
Vegetable poisons, Dr. Pickells on, 44
Ventilation, McKinnell's, 294
Water in St. George's, Hanover Square, 16; supply of London, report on, 214
Work, fresh air, and exercise, 251
Workhouses and the medical officers of health, 216
Working classes, city dwellings for, 296

### TRANSACTIONS

OF THE

### EPIDEMIOLOGICAL SOCIETY

### OF LONDON

FOR THE YEAR 1857.

LONDON:
T. RICHARDS, 37, GREAT QUEEN STREET

M.DCCC.LVII.

### NOTES AND CORRESPONDENCE.

The Editor is anxious to give free scope to the expression of opinion, but does not hold himself responsible for the opinions of those of his contributors who attach their names to their papers.

The Queen's Hospital, Birmingham.—We have received many communications on the subject of the controversy now going on regarding the election of surgeon to this Hospital. Mr. Joseph Sampson Gamgee competed for this appointment, and on the ground of pure merit obtained it by the conscientious voice of the Professors of the Queen's College. Against this sterling recognition of simple scientific claims, ignorance—there is no other word which expresses the fact—backed up by a fair amount of jealous selfishness, has raised its churlish, idiot, grinning face. We are not much concerned with surgical disputes; and, as our readers know, we often cross a lance with Mr. Gamgee. But we can afford to give to a friendly rival his due meed of honour, and to say for him that, as in these days the Hygiene of hospitals is a matter of far greater moment than any amount of skilful flesh-hewing or blood-drawing, so could the two great secrets of surgical success, hygienic principles and operative skill, be entrusted to no more able or energetic man than Mr. Gamgee. Moreover, as the advocates of right against might, we are bound to his cause, and believe and hope that the common sense of the Birmingham folk will ultimately settle the question in favour of knowledge, justice, and liberality. Otherwise, what shall we close-borough Londoners think of Birmingham talk about progress, and all that drug-in-the-market verbiage?

*Apropos* to this controversy, it recalls to our mind an incident, showing at one and the same time an analogy and a difference between Metropolitan and Provincial tactics. A medical appointment was open for competition a few years since in our town. The electors had insecurely, or rather hastily, pronounced that there should be no favour, that merit should rule the day. There were many competitors, but ultimately the choice rested on two or three. One candidate, by testimonials and amount of labour in the department, stood the acknowledged first on such estimate; one or two other candidates stood first on local influence grounds. What must the council do in this dilemma? Refer the matter to a committee? Certainly. The committee met; it laid no claim to sapiency, but to a great amount of decision. A straightforward John Bull tradesman opened the debate. "Mr Chairman, don't let us go to the thing like a lot of humbugs—let's be honest, and satisfy everybody. Honesty is the best policy, sir. There's no doubt that Leydenjar, with the long titles and testimonials, is the best man, and knows as much about the duties as any man can; but we want somebody else, isn't that the fact (hear, hear). Well then, if we want somebody else, let's have him (cheers); and this is the resolution I have to propose:—'Your committee is of opinion, that as the parish is very small, there can be no necessity for a scientific officer: they think an ordinary practitioner will answer every purpose.'" It is the fact that this resolution, accurate in essence if not in the exact words, ruled the election, and this so admirably, that when the exploded Leydenjar met his successful rival, they shook hands as friendly as ever. The one was tacitly satisfied with the compliment to his science, thê other with the compliment to his pocket and local reputation, while many a hearty laugh did the proceedings excite amongst the science men about town. To the progressive and knowledge loving people of Birmingham as a whole, we would be abashed to offer such a Metropolitan incident as a pattern, although, to such few of them as "for á pretence make long prayers," it deserves recommendation, as being at least innocent, straightforward, and honest.

We abstain, according to our custom, from entering into any personalities on this topic, except in so far as this is personal;—that we hold in the profoundest admiration the enlightened and manly conduct of certain gentlemen, with whom we are equally unacquainted, viz.: the Reverend Chancellor Law, Mr. Sands Cox, Dr. Birt Davies, and the other Professors. In every sense, these are gentlemen of Birmingham, and of England.

The Editor has once more the pleasure of returning his warmest thanks to his learned coadjutors for the ready support which they have rendered, and which enables him to add another volume to the Sanitary Literature of Great Britain.

M. Baudens. We regret much to have to announce the untimely death of our learned and distinguished brother, M. Baudens, Physician General of the French army, and Author of the "Medical Mission to the army of the East."

Dr. Richardson would be obliged if correspondents would kindly address their papers and books for review to his private residence, 12, Hinde Street, Manchester Square, W.

---

### BOOKS RECEIVED.

Aitken (W., M.D.) Handbook of the Science and Practice of Medicine. London and Glasgow: Richard Griffin & Co. 1858.

Beale (L.,M.D.) Archives of Medicine. London: Churchill, 1857.

Burt (J.G. M., M.D.) Letter to Viscount Palmerston on Medical Reform, 1857. [A spirited and pointed letter.]

Cockle (John, M.D.) Lecture Introductory to a Course of Pathology. London: T. Richards.

Fuller (H. W., M.D.) Advice to Medical Students; being the Address delivered at St. George's Hospital at the opening of the Session, October 1, 1857. London: Churchill.

Gamgee (J. Sampson). The Surgical Epoch: an Introductory Lecture to a Course of Clinical Surgery. Delivered in the Queen's College at Birmingham, November 16, 1857.

Huxley (J. E., M.D.) Annual Medical Report of the Kent Lunatic Asylum at Maidstone, for the year 1856-57.

Laycock (T., M.D) Correspondence and Statements regarding the Teaching of Clinical Medicine in the University of Edinburgh, 1855-7. With a Sequel. Edinburgh: 1857.

Miller (Jas., F.R.S.E.) Alcohol, its Place and Power. 12mo., Glasgow: Scottish Temperance League.

Radcliffe (J. N.) The Hygiene of the Turkish Army. From the Sanitary Review. London: Churchill 1858.

Statement of the Professors, and other Documents, relating to the Recent Election of Mr. Gamgee to the Office of Surgeon to the Queen's Hospital, Birmingham. 1857.

### DR. RICHARDSON.

### To New Subscribers to the Sanitary Review.

THE Publisher begs to intimate, that new Subscribers wishing to have the series of the Journal complete from the first number, can obtain sets of Vols. I and II, at Subscribers' prices, 10s. each, post free, by application to him, enclosing Post-office Order, payable at the Charing Cross Branch to THOMAS RICHARDS.

THE SANITARY REVIEW AND JOURNAL OF PUBLIC HEALTH is sold by all Booksellers, and is equally suited for the general and professional reader, for the scientific society, and for the private library.

London: T. RICHARDS, 37, Great Queen Street.

### NOTES AND CORRESPONDENCE.

The Editor is anxious to give free scope to the expression of opinion, but does not hold himself responsible for the opinions of those of his contributors who attach their names to the papers furnished by them. Friendship without fear, and freedom without strife, is the principle he would perpetuate in these pages.

*The Ozone Observations.* We have much pleasure in saying that a great many of the staff of Local Observers of disease have undertaken to add ozone observations. Some unlooked for delay has occurred in commencing, from the difficulties, 1st, of devising a plan of observation that shall be at once uniform and simple, and 2nd, of obtaining a constant supply of ozone papers, which shall yield correct results. In the dilemma, we have referred to Dr. Moffatt, who is at this time most kindly considering these points with us. As soon as the arrangements necessary are decided on, the papers and instructions will be issued according to Dr. Moffat's suggestions.

*The Tobacco Controversy.* We do not think there is sufficient evidence as yet collected to warrant any absolute conclusion as to the physiological effects of tobacco smoking. There are, of course, many cases in which the physician sees it necessary to order the discontinuance of the pipe or cigar, and amongst inveterate smokers, a class of symptoms may possibly be met with, which stand in the relation of effects to smoke as a cause. Whether smoking is a disgusting habit, is a question which each individual must answer according to his own taste. The scientific part we leave for the present to the more accurate researches of scientific men.

*Births and Deaths in Scotland.* The estimated population of Scotland to the middle of the year 1856, was 3,083,177 persons, including military. In 1856, the total number of births was 101,748, of which 52,301 were males, 49,447 were females. In the same period, the total number of deaths was 58,456, or 29,417 males, and 29,039 females. The excess of births over deaths thus numbered 43,292.

*Dr. Ayres*, the learned author of the first Original Paper in this Number, has left England for the Mauritius, where he now holds an important Government appointment in the Quarantine Establishment. No Government favour was ever better bestowed; we only regret that the scientific world of London has been robbed of a man whose whole life was a sacrifice to science, and whose further efforts must needs be crippled in the out of the way Mauritius. Are there no appointments at home for good men and enthusiasts?

We regret that the concluding part of Dr. Barker's valuable Essay is, by necessity, held over for next number, as well as several other communications of interest.

Erratum. In our number for October last, in the report on Liverpool, six deaths are stated to have occurred from destitution. In place of *six*, read *one*.

---

Subscribers are respectfully reminded that the Subscriptions to this Review are payable in advance. The Publisher would, therefore, be specially obliged if gentlemen who are in arrears for the past volume would forward such arrears to him as early as convenient by Post Office Order, or otherwise.

### BOOKS RECEIVED.

ALDIS (J. B., M.D., M.A.) Report on the Sanitary Condition of the Belgrave Sub-district in the Parish of St. George, Hanover Square. London: 1857.

BARKER (T. Herbert, M.D., F.R.C.S.) The Treatment of Fevers by Ventilation. 12mo. London: Richards. 1857.

BROWN (Isaac Baker, F.R.C.S.) On Scarlatina and its Treatment. 12mo. London: Churchill. 1857.

DANIEL (W. F., M.D.) On the Copals of Western Africa. Pamphlet. London: 1857.

DICKSON (Thomas, M.D.) Sixth Annual Report of the Manchester Royal Lunatic Hospital. Manchester: 1856.

FLEMING (Alexander, M.D.) On the Measle in the Pig, and the Wholesomeness as Food for Man of Measly Pork. Pamphlet. Dublin: McGlashan and Gill. 1857.

FULLER (William, M.D.) On Rheumatism, Rheumatic Gout, and Sciatica. Second Edition. London: Churchill. 1857.

[We regret that by an accident a notice of this excellent Work has been made to stand over for next Number.]

GLAISHER (James, F.R.S.) Meteorology. 12mo. An Excerpt Paper from Hughes' Reading Lesson Books. London: Longmans. 1857.

GLAISHER (James, F.R.S.) Report on the Meteorology of England for the Quarter ending December 31st, 1856. London. 1857.

GLAISHER (James, F.R.S.) Report on Meteorology of Scotland during the Quarter ending December 31st, 1856. London. 1857.

HUXLEY (J. E., M.D.) Annual Report on the Kent Lunatic Asylum. 1856.

MACKENZIE (Donaldson, Surgeon Dentist). On Caries of the Teeth and the Cure of Toothache without Extraction. 12mo. London: Churchill. 1855.

REPORT. The Sixth of the Council of the British Meteorological Society. London: 1857.

WILLIAMS (Thomas, M.D.) Report on the Copper Smoke and its Influence on Health, and the Industrial Diseases of Coppermen. London: Simpkin and Marshall. 1854.

---

### IN EXCHANGE.

Literary Gazette.
Psychological Journal.
British Medical Journal.
Ranking and Radcliffe's Abstract of the Medical Sciences.
Chemist.
Veterinarian.
Asylum Journal of Mental Science.
Quarterly Journal of the Chemical Society.
Scottish Review.
Edinburgh Medical Journal.
Glasgow Medical Journal.
Dublin Hospital Gazette.
American Journal of the Medical Sciences.
New York Journal of Medicine.
Montreal Medical Chronicle.
Charleston Medical Journal.
Gazette Médicale de Paris.
Aerzliches Intelligenz-Blatt.

